# The Administration of Anæsthetics to Children

**Published:** 1898-08-27

**Authors:** C. F. Marshall

**Affiliations:** late Anæsthetist to the Hospital for Sick Children, Great Ormond Street


### THE ADMINISTRATION OF ANÆSTHETICS TO CHILDREN.

By C. F. Marshall, M.D., F.R.C.S.Eng., late Anæsthetist to the Hospital for Sick Children, Great Ormond Street.

In spite of the large death-rate from chloroform administration, there is still a too prevalent idea that this drug is the best routine anæsthetic for children. The object of this article is to point out that chloroform, employed by itself, is never free from danger even in children, and that it can be replaced by safer methods with the same ease of administration. After considerable experience among children, I found a mixture of equal parts of chloroform and ether to be the best mixture. This is, I think, better than the A.C.E. mixture, as the alcohol in the latter is of doubtful use. The ether evaporates more quickly than the chloroform, and hence during the first few minutes the child is breathing chiefly ether, which tides over, so to speak, the initial depressing period of the chloroform—the most dangerous time during chloroform administration. It is no doubt true that children take chloroform well as a rule, but this is no argument in the face of the high death-rate from chloroform; and if we can use other safer methods, we are morally bound to do so.

Now let us consider the main differences between the anæsthetisation of children and adults, and the chief dangers to be encountered. (1) The corneal reflex, which is the guiding star of the anæsthetist in adults, is quite useless in children, being lost long before the patient is under. A better test is to sharply pinch the inner side of the thigh. If this produces no reflex contraction of the leg, the patient is fully under the anæsthetic. (2) All reflexes are more active in children than in adults, and movements often occur when consciousness is abolished. (3) Owing to this excess of reflex excitability, it is important not to begin the operation till the child is fully under. Many so-called deaths from anæsthetics are really due to the operator commencing too soon. (4) In many cases where elaborate antiseptic precautions are taken, the child is more or less enveloped in wet towels soaked in antiseptic solutions; these soon become cold, and the child is practically anæsthetised in a wet pack, which greatly adds to the shock of the operation and to the danger of the anæsthetic. If such a degree of antiseptic elaboration is required, the child should lie on a hot water bed, and the antiseptic cloths should be frequently charged with lotion kept hot. (5) In severe and prolonged operations the child's limbs should be wrapped as far as possible in flannel bandages, for the same reason as is mentioned above.

**The Choice of the Anæsthetic.**—For severe and prolonged operations ether alone should be used whenever possible. In children over eight years old this can be given by a Clover's inhaler, with a suitable mouthpiece. Younger children do well with the chloroform and ether mixture mentioned before. Very young babies, a few days or weeks old, can be anæsthetised by ether alone by the open method by means of a towel, the only objection to this being the large quantity of ether wasted. I have used this method several times for cases of circumcision, where there is danger of shock in giving chloroform alone.

For small operations and for the extraction of teeth, nitrous oxide can be given to older children. Bromide of ethyl is also useful where only two minutes' anæsthesia is required. Pharyngeal adenoids, if not too large, can be removed under bromide of ethyl, but severe cases require a longer period of anæsthesia. Cases of operation for cleft palate and other operations on the mouth and throat should be got under with ether, and kept under anæsthesia by the chloroform and ether mixture administered with a Junker's inhaler.

